# Depression-Burnout Overlap in Physicians

**DOI:** 10.1371/journal.pone.0149913

**Published:** 2016-03-01

**Authors:** Walter Wurm, Katrin Vogel, Anna Holl, Christoph Ebner, Dietmar Bayer, Sabrina Mörkl, Istvan-Szilard Szilagyi, Erich Hotter, Hans-Peter Kapfhammer, Peter Hofmann

**Affiliations:** 1 Department of Psychiatry and Psychotherapeutic Medicine, Medical University of Graz, Graz, Austria; 2 Department of Anesthesiology and Intensive Care Medicine, Medical University of Graz, Graz, Austria; 3 Arge Burnout, Graz, Austria; TNO, NETHERLANDS

## Abstract

**Background:**

Whether burnout is a distinct phenomenon rather than a type of depression and whether it is a syndrome, limited to three “core” components (emotional exhaustion, depersonalization and low personal accomplishment) are subjects of current debate. We investigated the depression-burnout overlap, and the pertinence of these three components in a large, representative sample of physicians.

**Methods:**

In a cross-sectional study, all Austrian physicians were invited to answer a questionnaire that included the Major Depression Inventory (MDI), the Hamburg Burnout Inventory (HBI), as well as demographic and job-related parameters. Of the 40093 physicians who received an invitation, a total of 6351 (15.8%) participated. The data of 5897 participants were suitable for analysis.

**Results:**

Of the participants, 10.3% were affected by major depression. Our study results suggest that potentially 50.7% of the participants were affected by symptoms of burnout. Compared to physicians unaffected by burnout, the odds ratio of suffering from major depression was 2.99 (95% CI 2.21–4.06) for physicians with mild, 10.14 (95% CI 7.58–13.59) for physicians with moderate, 46.84 (95% CI 35.25–62.24) for physicians with severe burnout and 92.78 (95% CI 62.96–136.74) for the 3% of participants with the highest HBI_sum (sum score of all ten HBI components). The HBI components *Emotional Exhaustion*, *Personal Accomplishment* and *Detachment* (representing depersonalization) tend to correlate more highly with the main symptoms of major depression (sadness, lack of interest and lack of energy) than with each other. A combination of the HBI components *Emotional Exhaustion*, *Helplessness*, *Inner Void* and *Tedium* (adj.R^2^ = 0.92) explained more HBI_sum variance than the three “core” components (adj.R^2^ = 0.85) of burnout combined. Cronbach’s alpha for *Emotional Exhaustion*, *Helplessness*, *Inner Void* and *Tedium* combined was 0.90 compared to α = 0.54 for the combination of the three “core” components.

**Conclusions:**

This study demonstrates the overlap of burnout and major depression in terms of symptoms and the deficiency of the three-dimensional concept of burnout. In our opinion, it might be preferable to use multidimensional burnout inventories in combination with valid depression scales than to rely exclusively on MBI when clinically assessing burnout.

## Introduction

Various studies have reported on the prevalence and potential causes of depression in physicians [1–4]. According to these studies, physicians have an increased risk of depressive symptoms compared to the general population [5]. One fatal consequence of this burden is the high prevalence of suicides among physicians, with an aggregate suicide rate ratio of 1.41 for male and 2.27 for female physicians, compared to the general population [6]. Both social and personality factors influence depressive symptoms in physicians. An association between depression in physicians and low social support from friends and family has been reported in previous studies [4, 7]. Married physicians have a lower risk of moderate or severe depression scores compared to their unmarried colleagues [1]. According to a Norwegian prospective study, neuroticism is a predictor of suicidal ideation among young physicians [8]. Besides personality factors and family stressors, work-related factors are also relevant, as occupational stress is associated with depression among physicians [2, 4, 9]. Physicians face occupational stressors such as long working hours, sleep deprivation, the demands of a high degree of professionalism and responsibility for patients, daily conflicts between ethical values and economic targets as well as the risk of medical errors and malpractice suits [4, 9, 10]. In recent decades, physicians’ work for their patients has become more challenging because of increased workload, increased administrative responsibilities, and decreased autonomy [10, 11].

In light of the foregoing considerations, physicians are ideally suited for studies on the relationship between depression and burnout. According to commonly accepted definitions, burnout is a persistent negative work-related state of mind that is defined by three components: emotional exhaustion, depersonalization and a low sense of personal accomplishment [12, 13]. As opposed to major depression, which affects all aspects of a patient´s life, burnout is considered a distinct work-related syndrome [14]. According to many studies, burnout is widespread among physicians [15–20]. In their study “Burnout and satisfaction with work-life balance among US physicians relative to the general US Population”, Shanafelt et al. revealed that physicians are much more likely to experience symptoms of burnout (37.9% vs 27.8%) and to be dissatisfied with their work-life balance (40.2% vs 23.2%) than other occupational groups [16]. A longitudinal study revealed that the prevalence of physician burnout increased after implementation of new health care policies in the United Kingdom [21].

What has led researchers to believe there is an overlap between burnout and depression? Since the concept of burnout was first introduced in 1974 by Freudenberger, the overlap between burnout and depression has been evident; as Freudenberger stated, a person suffering from burnout “acts and seems depressed” (p.161) [22]. Many studies have shown a positive correlation between burnout and depression in many occupational groups and by using different measures of depression and burnout [13, 23–25]. Furthermore, feelings of helplessness, hopelessness and powerlessness are found both in individuals with depression and burnout [23, 26, 27]. Individuals with a high degree of burnout are also likely to fulfill the diagnostic criteria of major depression [28, 29].

Some years ago, many authors agreed that burnout and depression conceptually overlap, but are not completely redundant [30–32]. The hypothesis that burnout is distinct from depression was supported by the finding that questionnaire items measuring burnout and depression do not assess the same factor, according to confirmatory factor analyses [23, 31, 32]. Nevertheless, the question whether burnout is a distinct phenomenon rather than a type of depression is the subject of current debate [23, 33, 34]. For instance, the validity of factoring studies is limited by the difference of time frames and response alternatives between the items of burnout and depression inventories [23, 31]. Furthermore, a longitudinal study with a person-centered approach revealed that symptoms of burnout and depression clustered and developed together, leading the authors to conclude that “burnout could be viewed as equivalent to depressive symptoms in work life” (p.35)[34].

Based on the initial, three-dimensional definition of burnout, the Maslach Burnout Inventory (MBI) largely dominates and shapes burnout research [13, 23]. By the end of the 1990s, more than 90% of the journal articles on burnout had used the MBI [13]. This is problematic as the MBI is “neither grounded in firm clinical observation nor based on sound theorizing” (p.3) [35]. In many studies, the emotional exhaustion dimension of the MBI is more strongly related to depression than to the other two components of burnout [23, 33, 36–41]. In his recently published review of 92 studies on the burnout-depression overlap, Bianchi concludes “that the distinction between burnout and depression is conceptually fragile” (p.28) [23]. The author suggests questioning the pertinence of the dimensions that have been initially chosen to define burnout as a syndrome and using conservative cutoffs for burnout in order to compare fully developed burnout with major depression [23]. Using a conservative cut-off, only 3% of the participants in his study on the burnout-depression overlap in French teachers met the inclusion criteria for severe burnout [33].

Despite the large literature on depression and burnout in physicians, to our knowledge, no study has evaluated the depression-burnout overlap in physicians across all specialties. Furthermore, until now, data on the prevalence of major depression and symptoms of burnout in Austrian physicians have been lacking. The main aim of our study was to investigate the depression-burnout overlap and the pertinence of the three components that define burnout as a syndrome in a large, representative cohort of physicians. First, we hypothesized that major depression and symptoms of burnout are widespread among Austrian physicians. Here we examined the prevalence of major depression and burnout symptoms. Second, we investigated the relationship between major depression and burnout. We hypothesized that depressive symptoms increase gradually between mild burnout, moderate burnout, severe burnout and major depression. According to our third, exploratory aim, we evaluated the pertinence of the three components that define burnout as a syndrome. We hypothesized that compared to the established combination of emotional exhaustion, depersonalization, and low sense of personal accomplishment, other symptoms that frequently occur in individuals suffering from burnout, load on the same factor and are better suited for the prediction of burnout. Therefore, we used the Hamburg Burnout Inventory (HBI) which considers a broader scope of burnout symptoms [42].

## Materials and Methods

### Participants and data collection

In a cross-sectional study, all physicians working in Austria were invited to answer a questionnaire which included the Major Depression Inventory (MDI) [43], the Hamburg Burnout Inventory (HBI) [42], as well as questions on demographic and job-related parameters (age, gender, partnership status, employment status, job function, and night hospital duty). The study was approved by the Ethics Committee of the Medical University of Graz (EK-Nr.: 23–042 ex 10/11). Physicians were invited to take part in our survey via announcements in the journal of the Austrian Medical Chamber. This journal is published monthly and distributed to all physicians working in Austria. Announcements were printed in three consecutive issues between November 2010 and February 2011, and physicians were given a link to the online questionnaires and necessary access information. Of the 40093 physicians who received an invitation, a total of 6351 physicians participated. Only participants who answered both the MDI and the HBI completely were included for further statistical analysis. Furthermore, we excluded double-entries and test-entries. In the end, we analyzed the data of 5897 participants. The data regarding “all Austrian physicians” for January 2011 was obtained from the Austrian Medical Chamber. The response rate was at a minimum 15.8%, as it was not possible to determine how many physicians read the announcements. The participants, however, are representative of all Austrian physicians with respect to age (χ²[1] = 0.44, n.s.), gender (χ²[1] = 0.12, n.s.), and employment status (χ²[2] = 0.62, n.s., see **Table 1**). The physicians were informed that their participation was voluntary and the data would be kept anonymous. All physicians who answered the HBI received immediate feedback on their test results.

**Table 1:** Demographic and work-related information for participants compared to all Austrian physicians.

		Participants (n = 5897)	All physicians (n = 40093)
Age	Years M (SD)	44.4 (10.5)	46.3 (11.3)
	Younger than 45	*47*.*2*	*42*.*6*
	Older than 44	*52*.*8*	*57*.*5*
Gender	Men	*55*.*5*	*53*.*1*
	Women	*44*.*5*	*46*.*9*
Employment	Employed	*59*.*4*	*54*.*9*
	Private practice	*26*.*3*	*26*
	Both	*11*	*14*.*4*

Percentages in Italics.

### Major Depression Inventory (MDI)

Symptoms of depression were assessed using the MDI [43]. This self-report mood questionnaire, developed by Per Bech (Frederiksborg General Hospital) consists of 10 items: *Sadness*, *Lack of Interest*, *Lack of Energy*, *Lack of Self-Confidence*, *Bad Conscience*, *Taedium Vitae*, *Concentration Deficits*, *Changed Activity*, *Sleep Disturbances*, and *Changed Appetite*. Each question has to be answered in terms of the last two weeks on a 6-point Likert scale with response options ranging from “*at no time*” to “*all the time*”. In contrast to other self-report inventories, the MDI is generative for the diagnosis and estimation of symptom severity of clinical depression according to the ICD-10 and DSM-IV. The MDI has adequate internal and external validity in measuring the severity of clinical depression [44]. In their study on the sensitivity and specificity of the MDI for diagnosing major depression, Bech et al. used the Schedule for Clinical Assessment in Neuropsychiatry (SCAN) as an index of validity for the clinician’s DSM-IV and ICD-10 diagnosis of major (moderate to severe) depression [43]. The sensitivity and specificity of the MDI was assessed in a sample of 43 participants covering a spectrum of depressive symptoms. In this study, the MDI sensitivity varied between 0.86 and 0.92 and the specificity varied between 0.82 and 0.86. Cuijpers et al. evaluated the sensitivity, specificity, and psychometric qualities of the Dutch translation of the MDI in a consecutive sample of 258 psychiatric outpatients [45]. Of these patients, 120 had a mood disorder (70 major depression, 49 dysthymia). A total of 139 subjects had a comorbid axis-I diagnosis, and 91 subjects had a comorbid personality disorder. According to their statistics, Cronbach's alpha of the MDI was 0.89, and the correlation between the MDI and the depression subscale of the SCL-90 was 0.79 (p< 0.001). In our study, the internal consistency of the MDI was α = 0.91. According to the DSM-IV, a diagnosis of major depression requires a score on at least five of nine (items 4 and 5 are combined) items, to be scored as follows: the score on items 1–3 must be at least 4, and on all other items at least 3. Either item 1 or 2 must have a score of 4 or 5. When used as a measuring scale for the gradation of major depression according to DSM-IV, the 10 items are added, with a score range from 0 to 50. Mild depression is defined by an MDI_sum (sum score of all ten MDI items) of 20 to 24, moderate depression by an MDI_sum of 25 to 29, and severe depression by an MDI_sum of 30 or more.

### Hamburg Burnout Inventory (HBI)

The HBI was developed by Matthias Burisch (University of Hamburg) [42]. It is a 40-item self-rating instrument measuring ten components: *Emotional Exhaustion*, *Personal Accomplishment*, *Detachment*, *Depressive Reaction to Stress*, *Helplessness*, *Inner Void*, *Tedium*, *Inability to Unwind*, *Overtaxing Oneself*, and *Aggressive Reaction to Stress*. The items are scored on 7-point Likert scales from 1 (*entirely incorrect*) to 7 (*entirely correct*). Items include: “Occasionally I feel a strong aversion to my work “(*Tedium*) and “I am only satisfied with myself if I have given my best” (*Overtaxing Oneself*). Between 2006 and 2013 the HBI was available online on a Swiss website. The instrument was preferred to the Maslach Burnout Inventory due to its broader scope and because validity information and rough norms, based on German data, are provided [42]. Burisch assessed the validity of the HBI by correlating HBI component scores with peer ratings obtained from two peers. These correlations corrected for attenuation due to criterion unreliability, range from a low of 0.11 to a high of 0.59 and an average of 0.46 in two studies. Cronbach’s alpha varies from 0.56 to 0.89 with a mean of 0.75 (note that HBI scales are very short). In our study, the internal consistency of the HBI was α = 0.94. In interpreting the HBI, a component score in the lower half of the sample is considered normal. To estimate the severity of burnout symptoms, the HBI component scores are stratified by quartiles (and the highest decile) of the sample [46]. As burnout is not recognized as a distinct disorder, like all burnout inventories, the HBI is not a tool for diagnosing burnout but for quantifying burnout symptoms in individuals. We carried out a grading of burnout symptoms by comparing an individual’s HBI_sum with the data of approximately 22.000 persons of different professions and medical students who participated in other (not yet published) survey studies carried out by our research group. The stratification by quartiles and the highest decile was based on this 22.000 person sample. Inspired by Bianchis`study on the burnout-depression overlap in French teachers [33], we also determined the odds ratio of suffering from major depression for the 3% of physicians with the highest HBI_sum, given that the prevalence of severely burnt-out individuals is comparable in both professions [47].

## Results

### 1. Prevalence of major depression and symptoms of burnout

Major depression affected 10.3% (n = 607) of the physicians. Of these, 0.9% (n = 50) fulfilled the criteria of mild, 2.3% (n = 135) of moderate and 7.2% (n = 422) of severe major depression. The cut-off for burnout was exceeded by 50.7% (n = 2988) of the participants-where 28% (n = 1653) had a mild, 13.1% (n = 771) a moderate and 9.6% (n = 564) a severe degree of burnout symptoms.

By grading burnout symptoms with quartile/decile cut-offs of the HBI_sum (sum score of all ten HBI components), the means of all ten HBI components increased gradually with the burnout grade (see **Table 2**).

**Table 2:** HBI components means, separated by burnout grade.

	Emotional Exhaustion	Personal Accomplish-ment	Detachment	Depressive Reaction to Stress	Helpless-ness	Inner Void	Tedium	Inability to Unwind	Overtaxing Oneself	Aggressive Reaction to Stress
No Burnout	15.38	7.49	10.34	8.46	8.73	7.26	10.47	9.23	22.9	8.9
Mild Burnout	24.86	9.13	13.78	12.4	15.92	13.71	18.45	13.56	25.96	13.18
Moderate Burnout	28.48	10.24	16.09	14.45	19.56	18.22	23.31	15.62	27.61	15.27
Severe Burnout	31.68	11.79	19.05	16.44	23.17	22.46	28.21	17.78	29.42	17.39

**No Burnout** means an HBI_sum in the first or second quartile (a score of ≤ 144). **Mild Burnout** is characterized by an HBI_sum in the third quartile (a score between 145–178). Individuals with **Moderate Burnout** have an HBI_sum between the third quartile and ninth decile (a score between 179–200). **Severe Burnout** is characterized by an HBI_sum in the highest decile (a score of ≥ 201).

Physicians younger than 45 years had a significantly higher mean HBI_sum (M = 144.41, SD = 40.27) compared to their older colleagues (HBI_sum: M = 139.26, SD = 43.18, t [5884.75] = 4.74, p< 0.001). Physicians in training had a significantly higher mean HBI_sum (M = 145.77, SD = 39.46) than specialists and general practitioners (HBI_sum: M = 139.34, SD = 42.37, t [2733.08] = -5.17, p< 0.001). Female physicians had a significantly higher mean HBI_sum (M = 145.36, SD = 40.56) than male physicians (HBI_sum: M = 138.37, SD = 42.81, t [5877.54] = 6.46, p< 0.001).

An ordinal logistic regression on the effects of age and gender on burnout confirmed an interaction between age and gender: Younger (< 45 years) women (M = 0.82) differ from younger men (M = 0.72; Estimate: 0.37, SE = 0.07, Wald = 32.55, p< 0.05). Older (≥ 45 years) women (M = 0.79) differ from older men (M = 0.70; Estimate: 0.21, SE = 0.07, Wald = 8.9, p< 0.05).

We separated the sample into physicians unaffected by burnout and depression (**UA**), physicians suffering from major depression without suffering from burnout (**MD**), physicians suffering from burnout without suffering from major depression (**BO**), and physicians suffering from both, major depression and burnout (**MD/BO**).

A total of 1.3% (n = 76) of physicians were in group **MD**. Of them, 4% (n = 3) had mild, 10.6% (n = 8) moderate and 85.6% (n = 65) severe depression. **BO** consisted of 37.4% (n = 2205) of physicians. Of them, 62.7% (n = 1382) had mild, 26.1% (n = 576) moderate and 11.2% (n = 247) severe burnout. In **MD/BO** were 9% (n = 531) of physicians. Of them, 8.9% (n = 47) had mild, 23.9% (n = 127) had moderate and 67.2% (n = 357) severe depression; as for burnout, 19.2% (n = 102) suffered from mild, 27.1% (n = 144) from moderate and 53.7% (n = 285) from severe burnout. **UA** comprised 52.3% (n = 3085) of the participants.

### 2. Relationship between major depression and burnout

Of the depressed physicians, 87.5% (n = 531) also suffered from burnout symptoms. Of the physicians suffering from burnout symptoms, 26.2% (n = 783) were also affected by major depression. The proportion of depressed physicians increased with the burnout grade (mild: 6.8%, moderate: 20.2%, severe: 53.6%). Compared to physicians unaffected by burnout symptoms, the odds ratio of suffering from major depression was 2.99 (95% CI 2.21–4.06) for physicians with a mild degree of burnout symptoms, 10.14 (95% CI 7.58–13.59) for physicians with a moderate degree of burnout symptoms, 46.84 (95% CI 35.25–62.24) for physicians with a severe degree of burnout symptoms and 92.78 (95% CI 62.96–136.74) for the 3% of the participants with the highest HBI_sum. For the whole group of participants, a Spearman rank–order correlation revealed a high correlation between MDI_sum and HBI_sum (rs = 0.74, p< 0.001). In **BO** the correlation between MDI_sum and HBI_sum was moderate (rs = 0.47, p< 0.001). Means of MDI_sum increased with the degree of burnout symptoms (mild: M = 14.17 [SD = 6.60], moderate: M = 19.25 [SD = 7.07], severe: M = 23.77 [SD = 7.75]. In **MD** the correlation between MDI_sum and HBI_sum was high (rs = -0.59, p< 0.001). Means of HBI_sum decreased with grade of major depression: mild: M = 126 (SD = 2.00), moderate: M = 116.63 (SD = 10.62) and severe: M = 98.43 (SD = 24.22). In **MD/BO** the correlation was high (rs = 0.52, p< 0.001).

**Fig 1:**
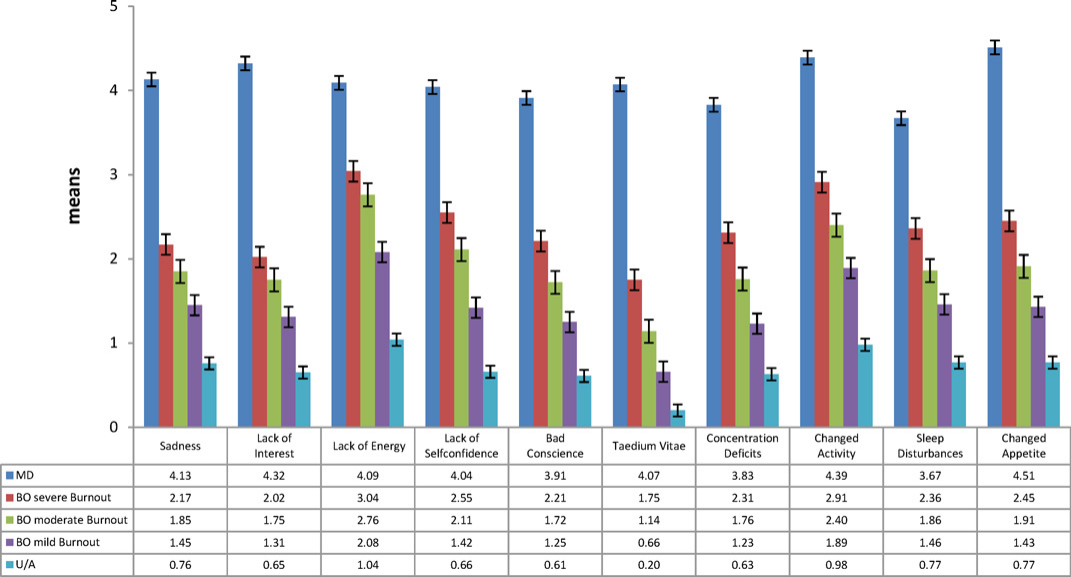
MDI item means across UA, mild BO, moderate BO, severe BO and MD. **UA** = physicians unaffected by burnout symptoms and major depression. **BO** = physicians suffering from burnout symptoms without suffering from major depression. Mild **BO** is characterized by an HBI_sum in the third quartile (a score between 145–178), individuals with moderate **BO** have an HBI_sum between the third quartile and ninth decile (a score between 179–200), and severe **BO** is characterized by an HBI_sum in the highest decile (a score of ≥ 201). **MD** = physicians suffering from major depression without suffering from burnout symptoms. T-tests revealed significant increases in all MDI item means across **UA**, mild **BO**, moderate **BO**, severe **BO** and **MD** (see **S1 Table**).

Mann-Whitney U tests revealed that the means of all ten MDI items increased gradually between unaffected, mildly burnt-out, moderately burnt-out, severely burnt-out and depressed physicians (p< 0.001; see **Fig 1**).

### 3. Evaluation of the three components that define burnout as a syndrome

A Spearman rank–order correlation revealed that *Emotional Exhaustion*, *Personal Accomplishment* and *Detachment* tend to correlate more highly with the main symptoms of major depression (*Sadness*, *Lack of Interest*, *Lack of Energy [48])* than with each other (see **Table 3**). These findings raise the question as to whether these three “core” components of burnout are suitable for determining burnout. A symptom gradation is possible for all ten components of the HBI. After ruling out collinearity between the HBI components (see **S2 Table**), we calculated a binary logistic regression to identify HBI components with the highest correlations with burnout (-2 Log-Likelihood = 2067.9, χ² = 6067.42, p <0.001; R² Nagelkerke = 0.86). In addition to the by far most suitable component *Emotional Exhaustion*, we identified *Helplessness*, *Inner Void* and *Tedium* as the HBI components with the highest correlations with HBI_sum and the highest odds ratio for burnout (HBI_sum≥ 145; see **Table 4**). In the next step, for examining best fitting models of burnout based on the study data, we performed a confirmatory factor analysis. Compared to the “core” components *Personal Accomplishment* and *Detachment*, the “identified” factors *Helplessness*, *Inner Void* and *Tedium* loaded on the same factor as *Emotional Exhaustion* (see **Table 5**). By calculating linear regressions, we confirmed that the “identified” components (i.e., *Emotional Exhaustion*, *Helplessness*, *Inner Void* and *Tedium*) combined can explain more of the HBI_sum variance than the three “core” components combined (see **Table 6**). Cronbach’s alpha for the four “identified components” combined was 0.90 compared to α = 0.54 for the combination of the three “core” components. According to a hierarchical linear regression, the addition of *Emotional Exhaustion* to *Helplessness*, *Inner Void* and *Tedium* exhibits better explained variance of HBI_sum (see **S3 Table**). In order to compare three component combinations of the “core” components and the “identified” new components, we finally performed a linear regression that excluded *Emotional Exhaustion*. Also *Helplessness*, *Inner Void* and *Tedium* combined explained more HBI_sum variance than the three “core” components combined (see **Table 6**) and had a significantly (Fishers Z = -12.86; p <0.001) higher correlation with HBI_sum (r = 0.94) than three “core” components combined (r = 0.91). Although the Spearman rank–order correlation revealed moderate to high correlations with the main symptoms of major depression, the correlations within *Helplessness*, *Inner Void* and *Tedium* were higher (see **Table 3**).

**Table 3:** Spearman–rank order correlations between HBI components and main symptoms of major depression.

	Emotional Exhaustion^a^	Personal Accomplishment^a^	Detachment^a^	Helplessness^a^	Inner Void^a^	Tedium^a^	Sadness^b^	Lack of Interest^b^
Personal Accomplishment^a^	*0*.*28*							
Detachment^a^	*0*.*43*	*0*.*25*						
Helplessness^a^	*0*.*76*	*0*.*41*	*0*.*48*					
Inner Void^a^	*0*.*73*	*0*.*39*	*0*.*56*	*0*.*78*				
Tedium^a^	*0*.*72*	*0*.*40*	*0*.*51*	*0*.*70*	*0*.*69*			
Sadness^b^	*0*.*52*	*0*.*30*	*0*.*34*	*0*.*60*	*0*.*59*	*0*.*49*		
Lack of Interest^b^	*0*.*50*	*0*.*31*	*0*.*40*	*0*.*53*	*0*.*58*	*0*.*54*	*0*.*60*	
Lack of Energy^b^	*0*.*65*	*0*.*29*	*0*.*35*	*0*.*59*	*0*.*60*	*0*.*53*	*0*.*63*	*0*.*64*

^a^ = HBI components

^b^ = MDI items.

**Table 4:** Odds Ratio for burnout (HBI_sum≥ 145), predicted by the components of the HBI.

**HBI components**	**Odds Ratio**
Emotional Exhaustion	*13*.*89*
Personal Accomplishment	*2*.*54*
Detachment	*4*.*86*
Depressive Reaction to Stress	*3*.*47*
Helplessness	*9*.*45*
Inner Void	*8*.*95*
Tedium	*8*.*27*
Inability to Unwind	*4*.*42*
Overtaxing Oneself	*5*.*23*
Aggressive Reaction to Stress	*2*.*54*

**Table 5:** Confirmatory Factor Analysis of the HBI components.

HBI components	Factor		
	1	2	3
Emotional Exhaustion	*0*.*753*	*0*.*432*	*0*.*119*
Personal Accomplishment	*0*.*312*	*-0*.*108*	*0*.*563*
Detachment	*0*.*533*	*0*.*111*	*0*.*249*
Depressive Reaction to Stress	*0*.*343*	*0*.*597*	*0*.*521*
Helplessness	*0*.*695*	*0*.*414*	*0*.*387*
Inner Void	*0*.*741*	*0*.*272*	*0*.*326*
Tedium	*0*.*768*	*0*.*173*	*0*.*271*
Inability to Unwind	*0*.*422*	*0*.*579*	*0*.*123*
Overtaxing Oneself	*0*.*091*	*0*.*678*	*-0*.*141*
Aggressive Reaction to Stress	*0*.*498*	*0*.*456*	*0*.*268*

**Table 6:** Linear Regression: Explained variance of HBI_sum by different combinations of HBI components.

	R²	adj. R²	ß	t	r	sr
“Core” components	*0*.*85*	*0*.*85*				
Emotional Exhaustion			*0*.*70*	*121*.73*	*0*.*86*	*0*.*85*
Personal Accomplishment			*0*.*17*	*30*.90*	*0*.*41*	*0*.*38*
Detachment			*0*.*27*	*47*.18*	*0*.*62*	*0*.*52*
Identified components	*0*.*92*	*0*.*92*				
Emotional Exhaustion			*0*.*30*	*49*.76*	*0*.*86*	*0*.*54*
Helplessness			*0*.*33*	*49*.97*	*0*.*89*	*0*.*55*
Inner Void			*0*.*24*	*38*.55*	*0*.*85*	*0*.*45*
Tedium			*0*.*22*	*38*.62*	*0*.*82*	*0*.*45*
Identified components without Emotional Exhaustion	*0*.*89*	*0*.*89*				
Helplessness			*0*.*44*	*60*.34*	*0*.*89*	*0*.*62*
Inner Void			*0*.*30*	*41*.80*	*0*.*85*	*0*.*48*
Tedium			*0*.*31*	*48*.58*	*0*.*82*	*0*.*54*

β = standardized regression coefficient; R² = explained variance; sr = semi– partial correlation

* = p< 0.001.

## Discussion

The main aim of our study was to investigate the depression-burnout overlap and the pertinence of the three components that define burnout as a syndrome in a large, representative cohort of physicians. We found that major depression and symptoms of burnout are widespread in Austrian physicians. Of the participants, 10.3% were affected by major depression and 50.7% exceeded the cut-off for burnout, used in our study. Our data confirm our hypothesis that depressive symptoms increase gradually between unaffected, mildly burnt-out, moderately burnt-out, severely burnt-out and depressed physicians. In sum, the current study emphasizes the overlap of burnout and major depression in terms of symptoms and the deficiency of the three-dimensional concept of burnout. To our knowledge, this is the first study on the depression-burnout overlap in physicians across all the specialties. Furthermore, this is the first study on the prevalence of major depression and burnout symptoms in Austrian physicians.

This study contributes in three ways to the existing literature. First, we confirmed our hypothesis that major depression and burnout symptoms are widespread among Austrian physicians. The participants had a more than twofold risk of major depression in relation to the global point prevalence of major depressive disorder [5]. Our study results, suggesting that potentially one in two Austrian physicians is affected by symptoms of burnout, are in accordance with a national study on burnout in U.S. physicians [16]. The fact that older physicians had lower burnout and depression scores is consistent with previously reported data [1, 4, 16, 19, 49]. One factor driving these differences may be young physicians’ high exposure to occupational stressors [50]. Physicians in training have to cope with “intense work demands, limited control and a high degree of work-home interference” (p.2880) [50]. Our finding that women had a significantly higher mean MDI_sum agrees well with the higher prevalence of major depression in female physicians, compared to their male colleagues [1]. The finding that women also had higher burnout scores is consistent with the observation that dissatisfaction with work-life balance is common in female physicians [51–53]. One explanation may be that women often assume greater responsibility at home. Summing up, our data identify a problem for both physicians and patients in Austria. Additional research on preventive and interventional measures against burnout and depression in physicians is required.

Second, this study demonstrates the particular importance of depressive symptoms in the burnout process. Our data are consistent with earlier findings that symptoms of burnout und depression are highly correlated and the risk of major depression increases with the grade of burnout [29, 33, 34]. Similar to the Finnish “Health 2000 study”, about half of those with severe burnout were affected by major depression [29]. Without valid cut-offs for severe (clinically relevant) burnout, Bianchi suggests using conservative cut-offs for studies on the overlap of severe burnout and depression [23, 54]. Given Bianchis’ finding, that only 3% of the participants in his study of French teachers met the inclusion criteria for severe burnout by using a conservative cut-off [33], we decided to investigate the odds for major depression in the 3% of our participants with the highest HBI_sum. Our stunning result that these participants had 93-fold odds of major depression supports the view that clinically relevant burnout is likely a form of depression [28]. When comparing **UA**, **BO** and **MD**, the means of all ten depression items increased gradually across unaffected, mildly burnt-out, moderately burnt-out, severely burnt-out and depressed physicians. Thus, physicians who suffered from severe burnout had lower mean scores on all MDI items compared to their depressed colleagues. Nevertheless, although they did not meet the diagnostic criteria for major depression, their mean MDI_sum ranged from mild to moderate major depression, when using the MDI as a measuring (and not diagnostic, see Methods section) scale. In other words, half of the participants with severe burnout met the diagnostic criteria of major depression and the other half had on average a pronounced depressive symptomatology. This finding emphasizes the frequent occurrence of depressive symptoms in severe burnout. Depression has both reliable diagnostic criteria and highly effective treatment options in contrast to burnout [55, 56]. Therefore, our findings lend considerable support to the proposal that depression scales with clinically valid cutoffs should be used as complements to burnout questionnaires when clinically assessing burnout [54]. Interestingly, the HBI_sum decreased with the grade of major depression in **MD**. A possible explanation may be an association between the degree of depression and work absence due to illness. Participants who suffered from moderate or severe major depression may not have been working for some time and therefore not exposed to occupational stressors. As we did not ask about sick leave in our questionnaire, we can only speculate that more physicians in group **MD/BO** were working despite being depressed than their colleagues in group **MD**.

Third, our findings question the pertinence of the three “core” components that define burnout as a syndrome (i.e., emotional exhaustion, depersonalization and low personal accomplishment). On the one hand, in line with other burnout inventories, the *Emotional Exhaustion* component of the HBI was highly correlated to the HBI_sum [57, 58]. On the other hand, our data is consistent with previous studies, pointing out that emotional exhaustion is more strongly linked to major depression than to the other two core components of burnout [23, 33, 36–41]. *Emotional Exhaustion* was highly correlated with all three main symptoms of depression (sadness, lack of interest, and lack of energy). These findings suggest that both burnout and depression conceptually overlap in terms of emotional exhaustion. Furthermore, the *Detachment* and *Personal Accomplishment* components of the HBI that represent the other two core components of burnout had a lower impact on burnout risk when compared to most other HBI components. Our finding that *Personal Accomplishment* is the HBI component with the lowest impact on burnout risk is consistent with the view that it is questionable to link low personal accomplishment to burnout syndrome [41]. In addition to *Emotional Exhaustion*, we identified *Helplessness*, *Inner Void* and *Tedium* as the central HBI components. *Helplessness* comprises feelings of being trapped, perplexity and despondency. *Inner Void* describes feelings of numbness, emptiness and lifelessness. *Tedium* defines a state of reluctance and job-related aversion. A combination of *Helplessness*, *Inner Void* and *Tedium* explained more variance in the HBI_sum than the three “core” components combined. Our results suggest that for the assessment of burnout, *Emotional Exhaustion* should rather be combined with *Helplessness*, *Inner Void* and *Tedium than with Personal Accomplishment* and *Detachment*. We conclude that the three-dimensional definition of burnout is conceptually fragile. On the one hand it conceptually overlaps with major depression and on the other hand it is inadequate for detecting the broad scope of burnout symptoms. Therefore, we support Bianchi´s argument that burnout may be a conceptual chimera that is based on the initial three-dimensional definition of burnout that underpins the MBI`s monopoly in burnout research [13, 23]. In our opinion, it might be more favorable to use multidimensional burnout inventories in combination with valid depression scales when clinically assessing burnout than exclusively using the MBI.

We should discuss at least three limitations of our study. First, the response rate of at least 15.8% was low. As it was not possible to determine how many physicians read the announcements, this rate might be substantially higher. Besides, phone follow up of the original sample may have helped to determine what percent of physicians had an office policy of non-responding to surveys. In a study of Canadian physicians, over 36.3% had a non-response office policy to any survey [59]. Unfortunately, we did not take this into consideration. Respondents were also slightly younger than the total population sample and thus may differ in other demographic and job-related parameters that either were not measured in our study or were not accessible for the total population sample. Nevertheless, the participants reflected a representative sample of all Austrian physicians with respect to age, gender, and employment status.

Second, our data on the prevalence of burnout and depression must be interpreted with caution. We should be aware that burnout has no binding diagnostic criteria and clinically valid cut-offs [54]. In addition, the comparison of our data with previous studies is limited, as this is the first study on depression and burnout among physicians using the MDI and the HBI. We used the HBI because of its broader scope of burnout components compared to the MBI. It is also obvious that physicians with a higher degree of depression symptoms are oversampled in our study, as the vast majority of the depressed participants suffered from severe major depression. These physicians may not have been working for some time and therefore not exposed to occupational stresses. Consequently, it is reasonable that they had more time and a greater willingness to take part in a study on depression and burnout. On the other hand, in general, survey participants tend to have better health compared to nonparticipants [60].

Third, the results of self-reports on psychological symptoms as used in our study may be biased by overestimation of symptomatology, so our data have to be cautiously considered. It would be important for future research to replicate our findings by conducting diagnostic interviews.

Our study has essential strengths. All Austrian physicians, regardless of job function, specialization and occupational environment were invited to participate. The participants represented all Austrian physicians with respect to age, gender, and employment status. We used a depression inventory that is generative for the diagnosis and estimation of symptom severity of clinical depression according to the ICD-10 and DSM-IV. Finally, as mentioned above, the burnout inventory used in our study investigates a broader scope of burnout components than the three-dimensional MBI.

## Conclusion

Our study indicates that major depression and symptoms of burnout are widespread in Austrian physicians. Additional research on preventive and interventional measures against depression and burnout in physicians is required. Depressive symptoms are particularly important in the process of burnout as severe burnout is likely to be a form of depression and the means of all ten depression items increased gradually between unaffected, mildly burnt-out, moderately burnt-out, severely burnt-out and depressed physicians. Our data on correlations between burnout components and symptoms of major depression emphasizes that the three-dimensional definition of burnout is conceptually fragile. On the one hand, the definition conceptually overlaps with major depression and on the other hand, it is inadequate for the detection of the broad scope of burnout symptoms. In our opinion, it would be preferable to use multidimensional burnout inventories in combination with valid depression scales to clinically assess burnout rather than exclusively using the MBI. Further studies are needed to test this hypothesis.

## Supporting Information

S1 Dataset (SAV)

S1 Table T-test-statistics for MDI item means, comparing UA, mild BO, moderate BO, severe BO and MD. **UA** = physicians unaffected by burnout symptoms and major depression. **BO** = physicians suffering from burnout symptoms without suffering from major depression. Mild **BO** is characterized by an HBI_sum in the third quartile (a score between 145–178), individuals with moderate **BO** have an HBI_sum between the third quartile and ninth decile (a score between 179–200), and severe **BO** is characterized by an HBI_sum in the highest decile (a score of ≥ 201). **MD** = physicians suffering from major depression without suffering from burnout symptoms).
(DOCX)

S2 Table Variance inflation factors (VIFs) for the HBI components. (DOCX)

S3 Table Hierarchical linear regression of the “identified” HBI components. (DOCX)
