# Supplementary material for: Depression-Burnout Overlap in Physicians
Source: PLoS One. 2016 Mar 1;11(3):e0149913. doi: 10.1371/journal.pone.0149913 (PMC4773131; doi:10.1371/journal.pone.0149913)
Supplement: S1 Table — UA = physicians unaffected by burnout symptoms and major depression. BO = physicians suffering from burnout symptoms without suffering from major depression. Mild BO is characterized by an HBI_sum in the third quartile (a score between 145–178), individuals with moderate BO have an HBI_sum between the third quartile and ninth decile (a score between 179–200), and severe BO is characterized by an HBI_sum in the highest decile (a score of ≥ 201). MD = physicians suffering from major depression without suffering from burnout symptoms). (DOCX) [file pone.0149913.s002.docx]

**S1** **Table.** T-test-statistics for MDI item means, comparing **UA**, mild **BO**, moderate **BO**, severe **BO** and **MD**.

| **Group Comparison UA/mild BO** | |  |  |  |  |
| --- | --- | --- | --- | --- | --- |
|  |  |  |  |  |  |
|  | t | df | p | **UA** Mean (SD) | mild **BO** Mean (SD) |
| Sadness | *-25.10* | *2255.58* | *0.001* | *0.76 (0.73)* | *1.45 (0.88)* |
| Lack of Interest | *-22.21* | *2416.80* | *0.001* | *0.65 (0.86)* | *1.31 (0.96)* |
| Lack of Energy | *-29.30* | *2153.29* | *0.001* | *1.04 (0.90)* | *2.08 (1.16)* |
| Lack of Selfconfidence | *-21.54* | *2016.87* | *0.001* | *0.66 (0.83)* | *1.42 (1.18)* |
| Bad Conscience | *-18.42* | *1977.93* | *0.001* | *0.61 (0.80)* | *1.25 (1.17)* |
| Taedium Vitae | *-15.62* | *1770.87* | *0.001* | *0.20 (0.58)* | *0.66 (1.04)* |
| Concentration Deficits | *-17.55* | *2012.85* | *0.001* | *0.63 (0.81)* | *1.23 (1.16)* |
| Changed Activity | *-23.56* | *2209.95* | *0.001* | *0.98 (1.02)* | *1.89 (1.27)* |
| Sleep Disturbances | *-15.85* | *2037.53* | *0.001* | *0.77 (1.04)* | *1.46 (1.45)* |
| Changed Appetite | *-14.34* | *2225.40* | *0.001* | *0.77 (1.21)* | *1.43 (1.50)* |
|  |  |  |  |  |  |
|  |  |  |  |  |  |
| **Group Comparison mild/moderate BO** | | |  |  |  |
|  |  |  |  |  |  |
|  | t | df | p | mild **BO** Mean (SD) | moderate **BO** Mean (SD) |
| Sadness | *-8.79* | *42.30* | *0.001* | *1.45 (0.88)* | *1.85 (0.95)* |
| Lack of Interest | *-8.80* | *1035.29* | *0.001* | *1.31 (0.96)* | *1.75 (1.00)* |
| Lack of Energy | *-10.96* | *983.14* | *0.001* | *2.08 (1.16)* | *2.76 (1.29)* |
| Lack of Selfconfidence | *-10.75* | *971.27* | *0.00* | *1.42 (1.88)* | *2.11 (1.33)* |
| Bad Conscience | *-7.34* | *952.74* | *0.00* | *1.25 (1.17)* | *1.72 (1.35)* |
| Taedium Vitae | *-8.04* | *920.71* | *0.00* | *0.66 (1.04)* | *1.14 (1.25)* |
| Concentration Deficits | *-7.86* | *908.52* | *0.00* | *1.23 (1.16)* | *1.76 (1.42)* |
| Changed Activity | *-7.79* | *1025.98* | *0.00* | *1.89 (1.27)* | *2.40 (1.34)* |
| Sleep Disturbances | *-5.15* | *976.44* | *0.00* | *1.46 (1.45)* | *1.86 (1.62)* |
| Changed Appetite | *-6.10* | *989.69* | *0.00* | *1.43 (1.49)* | *1.91 (1.65)* |
|  |  |  |  |  |  |
|  |  |  |  |  |  |
| **Group Comparison moderate/severe BO** | | |  |  |  |
|  | t | df | p | moderate **BO** Mean (SD) | severe **BO** Mean (SD) |
| Sadness | *-4.32* | *821.00* | *0.00* | *1.85 (0.95)* | *2.17 (0.99)* |
| Lack of Interest | *-3.50* | *459.67* | *0.00* | *1.75 (1.00)* | *2.02 (1.02)* |
| Lack of Energy | *-2.72* | *429.04* | *0.01* | *2.76 (1.29)* | *3.04 (1.42)* |
| Lack of Selfconfidence | *-4.12* | *435.00* | *0.00* | *2.11 (1.33)* | *2.55 (1.44)* |
| Bad Conscience | *-4.42* | *423.87* | *0.00* | *1.72 (1.35)* | *2.21 (1.51)* |
| Taedium Vitae | *-5.77* | *407.30* | *0.00* | *1.14 (1.25)* | *1.75 (1.47)* |
| Concentration Deficits | *-4.74* | *428.75* | *0.00* | *1.76 (1.42)* | *2.31 (1.56)* |
| Changed Activity | *-4.80* | *437.16* | *0.00* | *2.4 (1.33)* | *2.91 (1.43)* |
| Sleep Disturbances | *-3.92* | *445.17* | *0.00* | *1.86 (1.62)* | *2.36 (1.71)* |
| Changed Appetite | *-4.14* | *445.37* | *0.00* | *1.91 (1.64)* | *2.45 (1.73)* |
|  |  |  |  |  |  |
|  |  |  |  |  |  |
|  |  |  |  |  |  |
| **Group Comparison severe BO/MD** | |  |  |  |  |
|  | t | df | p | severe **BO** Mean (SD) | moderate **BO** Mean (SD) |
| Sadness | *14.32* | *118.05* | *0.00* | *2.17 (0.99)* | *4.13 (1.06)* |
| Lack of Interest | *17.70* | *128.22* | *0.00* | *2.02 (1.02)* | *4.32 (0.98)* |
| Lack of Energy | *7.03* | *169.38* | *0.00* | *3.04 (1.42)* | *4.09 (1.04)* |
| Lack of Selfconfidence | *8.43* | *134.28* | *0.00* | *2.55 (1.44)* | *4.04 (1.31)* |
| Bad Conscience | *9.16* | *134.70* | *0.00* | *2.21 (1.51)* | *3.91 (1.38)* |
| Taedium Vitae | *11.66* | *120.60* | *0.00* | *1.75 (1.47)* | *4.07 (1.53)* |
| Concentration Deficits | *7.34* | *123.11* | *0.00* | *2.31 (1.56)* | *3.83 (1.59)* |
| Changed Activity | *10.51* | *191.23* | *0.00* | *2.91 (1.43)* | *4.39 (0.94)* |
| Sleep Disturbances | *6.04* | *129.24* | *0.00* | *2.36 (1.71)* | *3.76 (1.64)* |
| Changed Appetite | *13.17* | *226.37* | *0.00* | *2.45 (1.73)* | *4.51 (0.97)* |
|  |  |  |  |  |  |

**UA**= physicians unaffected by burnout symptoms and major depression. **BO**= physicians suffering from burnout symptoms without suffering from major depression. Mild **BO** is characterized by an HBI_sum in the third quartile (a score between 145-178), individuals with moderate **BO** have an HBI_sum between the third quartile and ninth decile (a score between 179-200), and severe **BO** is characterized by an HBI_sum in the highest decile (a score of ≥ 201). **MD**= physicians suffering from major depression without suffering from burnout symptoms.
