# Supplementary material for: Depression-Burnout Overlap in Physicians
Source: PLoS One. 2016 Mar 1;11(3):e0149913. doi: 10.1371/journal.pone.0149913 (PMC4773131; doi:10.1371/journal.pone.0149913)
Supplement: S2 Table — (DOCX) [file pone.0149913.s003.docx]

**S2 Table.** Variance inflation factors (VIFs) for the HBI components.

|  | |  |  |
| --- | --- | --- | --- |
|  |  | **Tolerance** | **VIF** |
|  | Emotional Exhaustion | *0.32* | *3.18* |
|  | Personal Accomplishment | *0.72* | *1.39* |
|  | Detachment | *0.63* | *1.59* |
|  | Depressive Reaction to Stress | *0.44* | *2.28* |
|  | Helplessness | *0.25* | *4.02* |
|  | Inner Void | *0.31* | *3.18* |
|  | Tedium | *0.38* | *2.67* |
|  | Inability to Unwind | *0.53* | *1.88* |
|  | Overtaxing Oneself | *0.71* | *1.42* |
|  | Aggressive Reaction to Stress | *0.50* | *2.02* |
