# Supplementary material for: Depression-Burnout Overlap in Physicians
Source: PLoS One. 2016 Mar 1;11(3):e0149913. doi: 10.1371/journal.pone.0149913 (PMC4773131; doi:10.1371/journal.pone.0149913)
Supplement: S3 Table — (DOCX) [file pone.0149913.s004.docx]

**S3 Table.** Hierarchical linear regression of the “identified” HBI components.

| Model |  | B | SE | Beta | t | p |
| --- | --- | --- | --- | --- | --- | --- |
| 1 |  | 62.80 | 0.60 |  | 105.26 | 0.00 |
|  | Helplessness | 5.99 | 0.04 | 0.89 | 146.10 | 0.00 |
| 2 |  | 55.2 | 0.50 |  | 110.40 | 0.00 |
|  | Helplessness | 4.18 | 0.05 | 0.62 | 90.78 | 0.00 |
|  | Tedium | 2.01 | 0.04 | 0.38 | 56.24 | 0.00 |
| 3 |  | 54.73 | 0.44 |  | 124.60 | 0.00 |
|  | Helplessness | 2.99 | 0.05 | 0.44 | 60.34 | 0.00 |
|  | Tedium | 1.6 | 0.03 | 0.31 | 48.58 | 0.00 |
|  | Inner Void | 1.95 | 0.05 | 0.30 | 41.80 | 0.00 |
| 4 |  | 44.62 | 0.42 |  | 105.99 | 0.00 |
|  | Helplessness | 2.22 | 0.04 | 0.33 | 49.97 | 0.00 |
|  | Tedium | 1.13 | 0.03 | 0.22 | 38.62 | 0.00 |
|  | Inner Void | 1.54 | 0.04 | 0.24 | 38.55 | 0.00 |
|  | Emotional Exhaustion | 1.55 | 0.03 | 0.30 | 49.76 | 0.00 |
